# Supplementary material for: Mechanism of ATP hydrolysis in the Hsp70 BiP nucleotide-binding domain
Source: Nat Commun. 2025 Jun 1;16:5086. doi: 10.1038/s41467-025-60343-x (PMC12126525; doi:10.1038/s41467-025-60343-x)
Supplement: Supplementary file 1 — Supplementary Information [file 41467_2025_60343_MOESM1_ESM.pdf]

Supplementary Information:

Mechanism of ATP hydrolysis in the Hsp70 BiP nucleotide binding domain

Guillaume Mas, Sebastian Hiller*

Affiliation

Biozentrum, University of Basel, Spitalstrasse 41, 4056 Basel, Switzerland

*correspondence to sebastian.hiller@unibas.ch

This file includes:

- Supplementary Tables 1–2
- Supplementary Figures 1–18

Supplementary Table 1. Kinetic parameters of reactions involved in the BiP NBD functional cycle determined by independent experiments.[a]

label	reaction	K_D [μM]	k_{on} [$mM^{-1}s^{-1}$]	k_{off} [s^{-1}]	k^*_{cat} [s^{-1}]
1^{MABA}	NBD·MABA-ATP ⇌ NBD + MABA-ATP	1 (ref. 66)	32 (ref. 66)	0.031 (ref. 66)	
2	NBD·ADP ⇌ NBD + ADP	1.6 ± 0.2 (ITC)			
2^{MABA}	NBD·MABA-ADP ⇌ NBD + MABA-ADP	1.3 ± 0.4 (ITC)		0.052 ± 0.004 (MABA-ADP)	
3	NBD·Pi ⇌ NBD + Pi	310 ± 40 (NMR)	5 ± 2 (EXSY)	1.32 ± 0.31 (EXSY)	
4	NBD·ADP·Pi ⇌ NBD·ADP + Pi	280 ± 50 (NMR)			
4^{MABA}	NBD·MABA-ADP·Pi ⇌ NBD·MABA-ADP + Pi	270 ± 40 (NMR)			
5	NBD·ADP·Pi ⇌ NBD·Pi + ADP	0.90 ± 0.10 (ITC)			
5^{MABA}	NBD·MABA-ADP·Pi ⇌ NBD·Pi + MABA-ADP	1.0 ± 0.1 2.0 (ITC)		0.0064 ± 0.0003 (MABA-ADP)	
cat	NBD·ATP → NBD·ADP·Pi				0.071 ± 0.009 (single turn-over)

[a]Experimental method used or reference is given in brackets for each value

Supplementary Table 2. Kinetic parameters of the BiP NBD functional cycle at standard buffer conditions and T = 37°C, with MABA-ATP instead of ATP.

Label	Reaction	K_D [µM]	k_{on} [mM^{-1} s^{-1}]	k_{off} [s^{-1}]	k_{cat} [s^{-1}]
1^{MABA}	NBD·MABA-ATP ⇌ NBD + MABA-ATP	0.95 ± 0.1	37 ± 6	0.035 ± 0.008	
2^{MABA}	NBD·MABA-ADP ⇌ NBD + MABA-ADP	1.2 ± 0.2	42 ± 5	0.051 ± 0.008	
3	NBD·Pi ⇌ NBD + Pi	280 ± 30	3.5 ± 0.5	1 ± 0.3	
4^{MABA}	NBD·ADP·PI ⇌ NBD·MABA-ADP + Pi	360 ± 20	0.12 ± 0.02	0.043 ± 0.008	
5^{MABA}	NBD·MABA-ADP·Pi ⇌ NBD·Pi + MABA-ADP	0.80 ± 0.1	7.5 ± 2	0.0060 ± 0.0008	
cat^{MABA}	NBD·MABA-ATP → NBD·MABA-ADP·Pi				0.036 ± 0.004

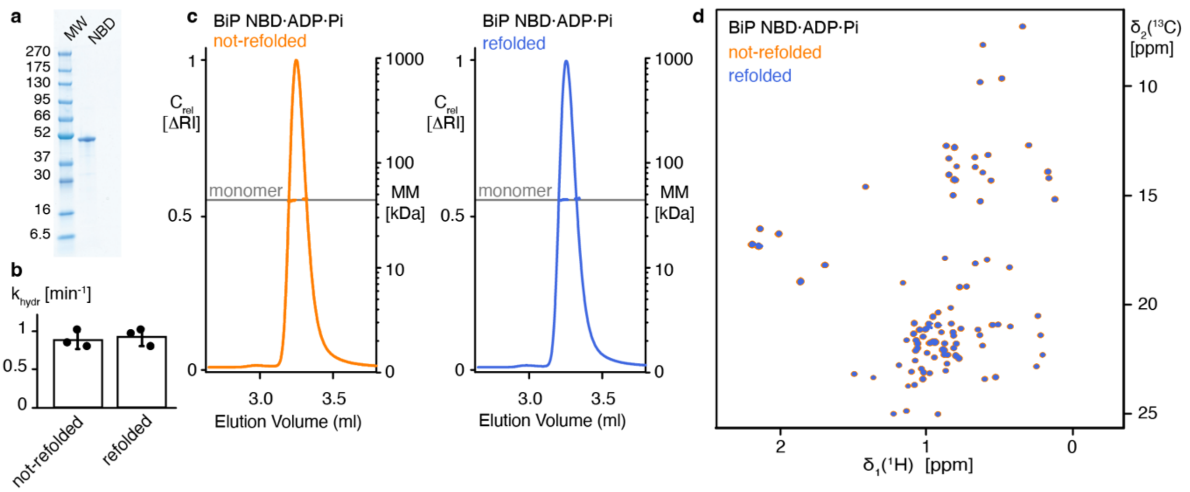

Supplementary Figure 1. Robustness of BiP NBD to refolding. a, SDS-PAGE of refolded BiP NBD along with a molecular weight marker (MW). **b**, ATPase activity of not-refolded and refolded BiP NBD, measured by NADH-coupled ATP assay. Data points represent three independent experiments. The bar represents mean and standard deviation. **c**, SEC elution profile (solid lines, left axis) and MALS apparent molecular mass (MM) (bold lines, right axis) at elution concentrations around 20 µM and a temperature of 25 °C (BiP NBD not-refolded (orange) and refolded (blue)). Gray horizontal lines indicate the expected molecular mass of the NBD monomer. **d**, Overlay of 2D [^{13}C,^{1}H]-TROSY spectra of methyl-labeled BiP NBD in presence of 5 mM ADP·Pi. Not-refolded, orange; Refolded, blue.

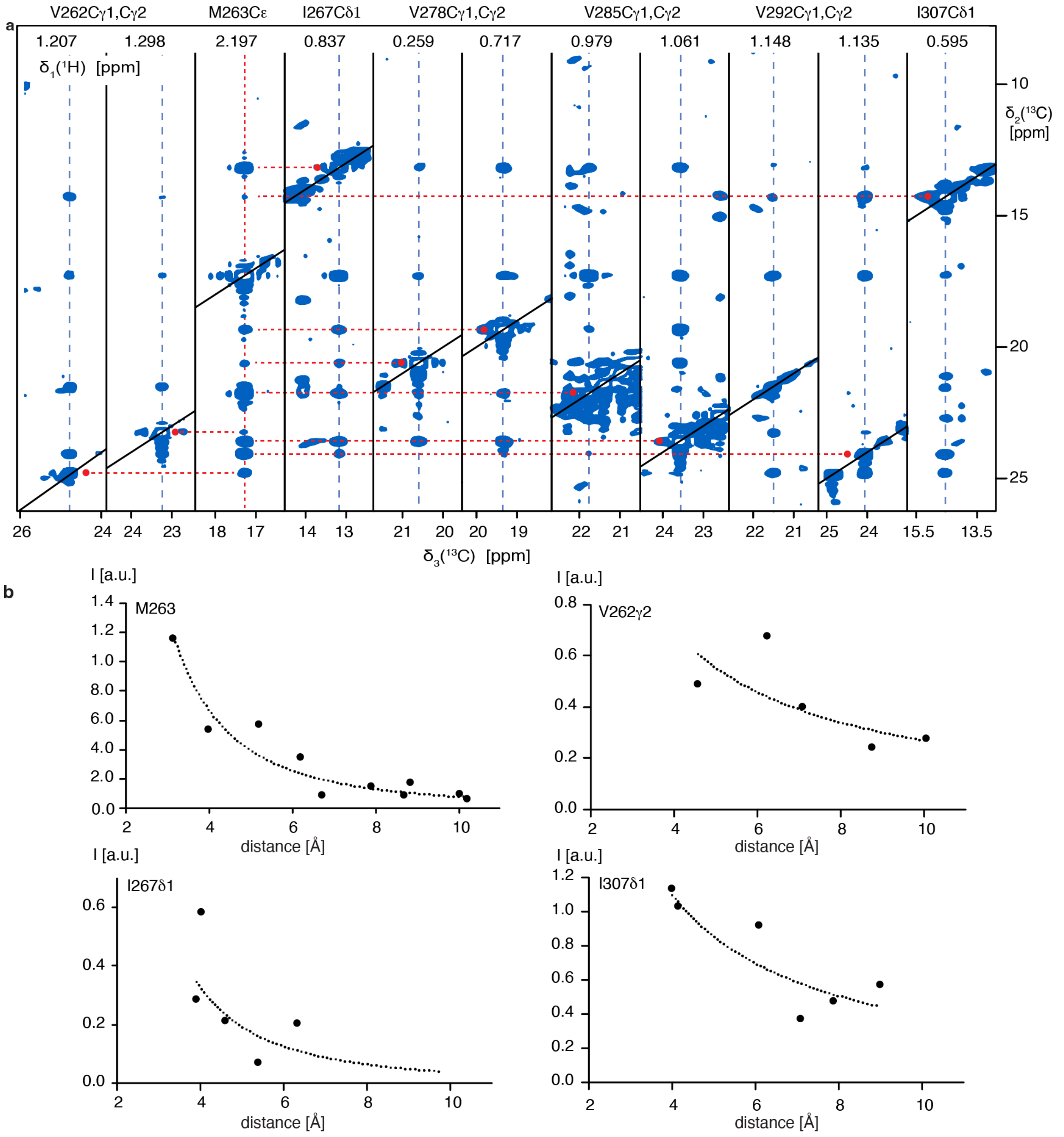

Supplementary Figure 2. Assignment of the ADP·Pi-bound state of BiP NBD. a, 2D strips of the 3D ^{13}C, ^{13}C-resolved [^{1}H, ^{1}H]-NOESY spectrum of methyl-labeled NBD in presence of 5 mM ADP and 5 mM Pi for selected residues. Assignment of significant NOE cross-peaks is indicated and connected by dashed pink lines. **b,** Cross peak intensity as a function of interatomic distance for the NOEs around methyl groups M263Cε, V262Cγ2, I267Cδ1 and I307Cδ1. Each black dot corresponds to an observed cross peak, plotted against the distance observed in crystal structure PDB 5EVZ. The black dashed line is an r^{-6} fit to the data.

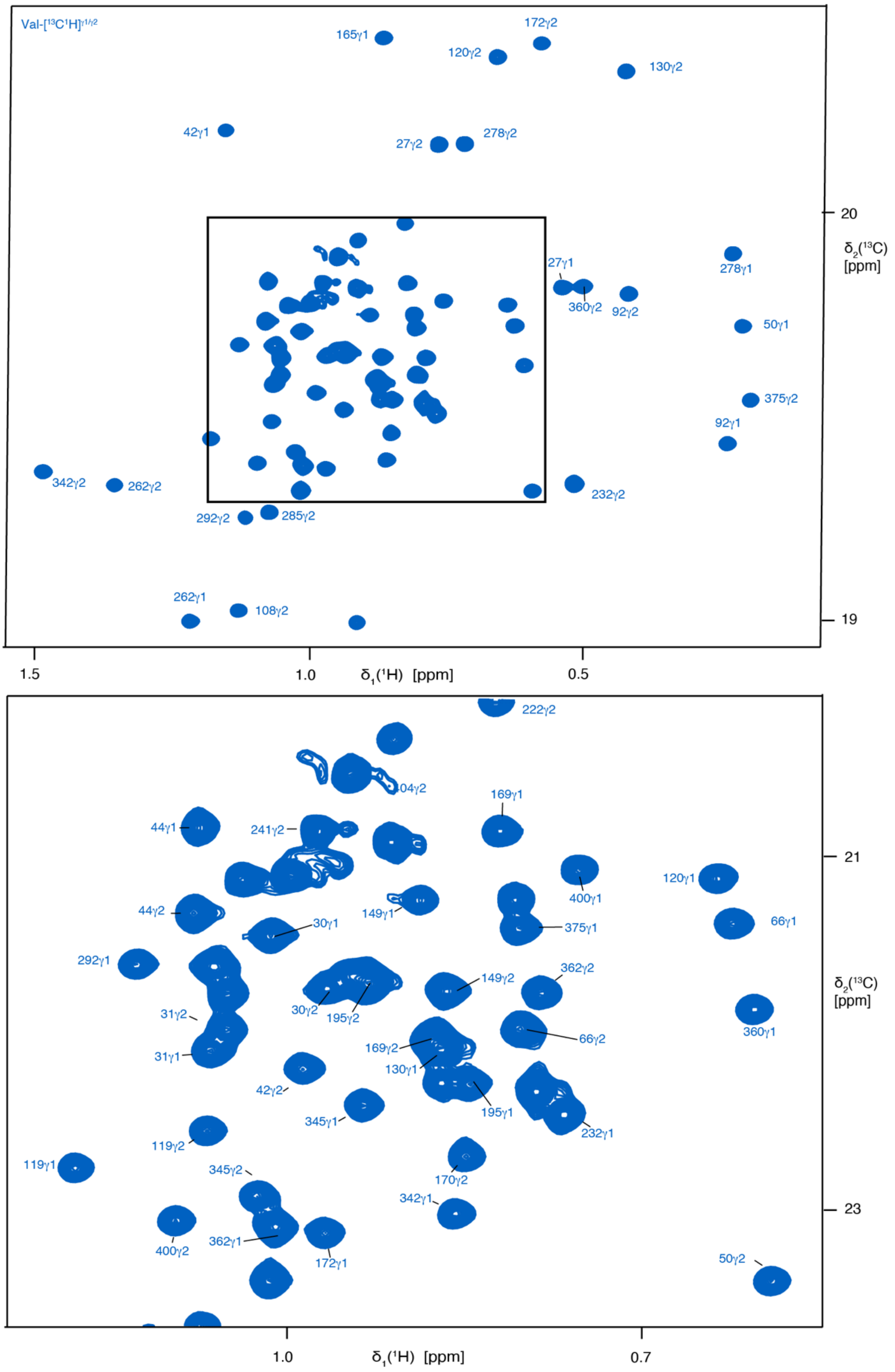

Supplementary Figure 3. Stereospecific assignment of BiP NBD Val-[^{13}C^{1}H]$^{\gamma1/\gamma2}$ in the ADP·Pi-bound state. 2D [^{13}C,^{1}H]-TROSY spectrum of methyl-labeled BiP in presence of 5 mM ADP·Pi. Sequence-specific resonance assignments are indicated.

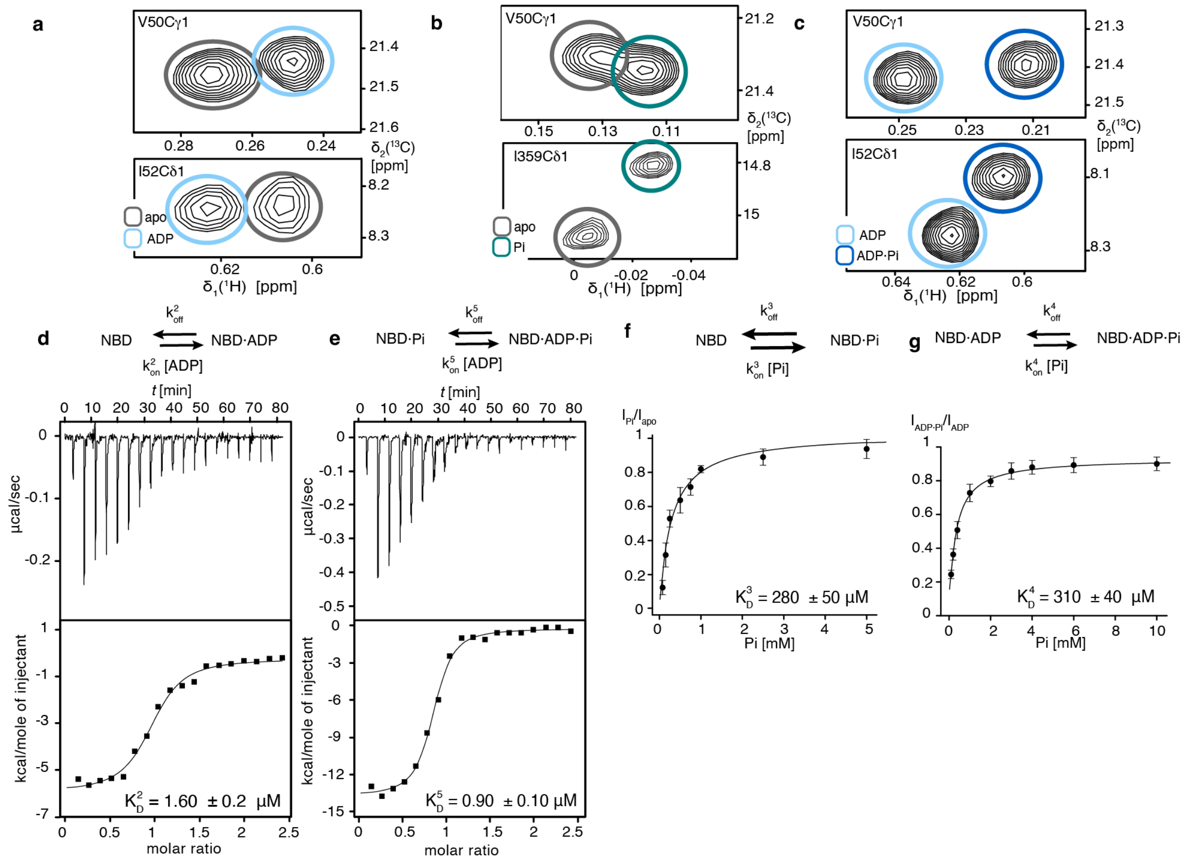

Supplementary Figure 4. Determination of dissociation constants. a-c, Selected sections of 2D [^{13}C,^{1}H]-methyl-TROSY spectra of residue Val50cγ1 for ADP (a), Pi (b), and ADP·Pi (c) showing the two NMR signals in slow exchange assigned to their corresponding states. Spectrum in (b) was recorded at 25°C as the protein stability is lower in the absence of nucleotide. **d,** ITC titration of 25 µM BiP NBD in the cell with 0.5 mM ADP in the syringe. **e**, ITC titration of 25 µM BiP NBD and 5 mM Pi in the cell with 0.5 mM ADP and 5 mM Pi in the syringe. **f**, NMR titration of 100 µM BiP NBD with increasing concentration of Pi. The Magnitude of normalized intensity the population of the NBD·Pi and NBD bound states are presented. **g**, NMR titration of 100 µM BiP NBD in the presence of 1 mM ADP with increasing concentration of Pi. The normalized intensities of the NBD·ADP·Pi and NBD·ADP bound state are presented. Data were fitted to extract the dissociation constant that are indicated next to the titration data for all the experiments.

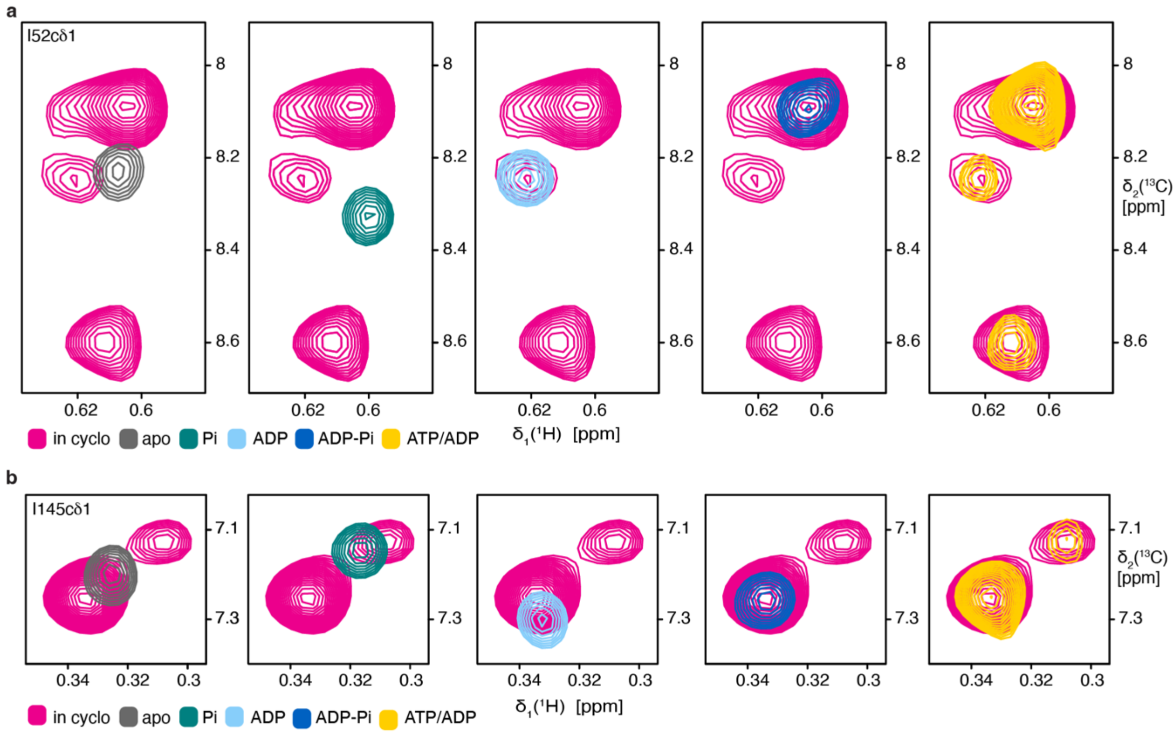

Supplementary Figure 5. Comparison of BIP NBD conformational states. a-b, Selected sections of 2D [^{13}C,^{1}H]-methyl-TROSY spectra of residue Ile52cδ1 (a) or Ile145cδ1 in-cyclo (b) (pink) compared to the equilibrium experiments of NBD apo (grey), NBD with 5 mM Pi (green), NBD with 5 mM ADP (light blue), NBD with 5 mM ADP·Pi (dark blue) and NBD with 5 mM ATP (yellow).

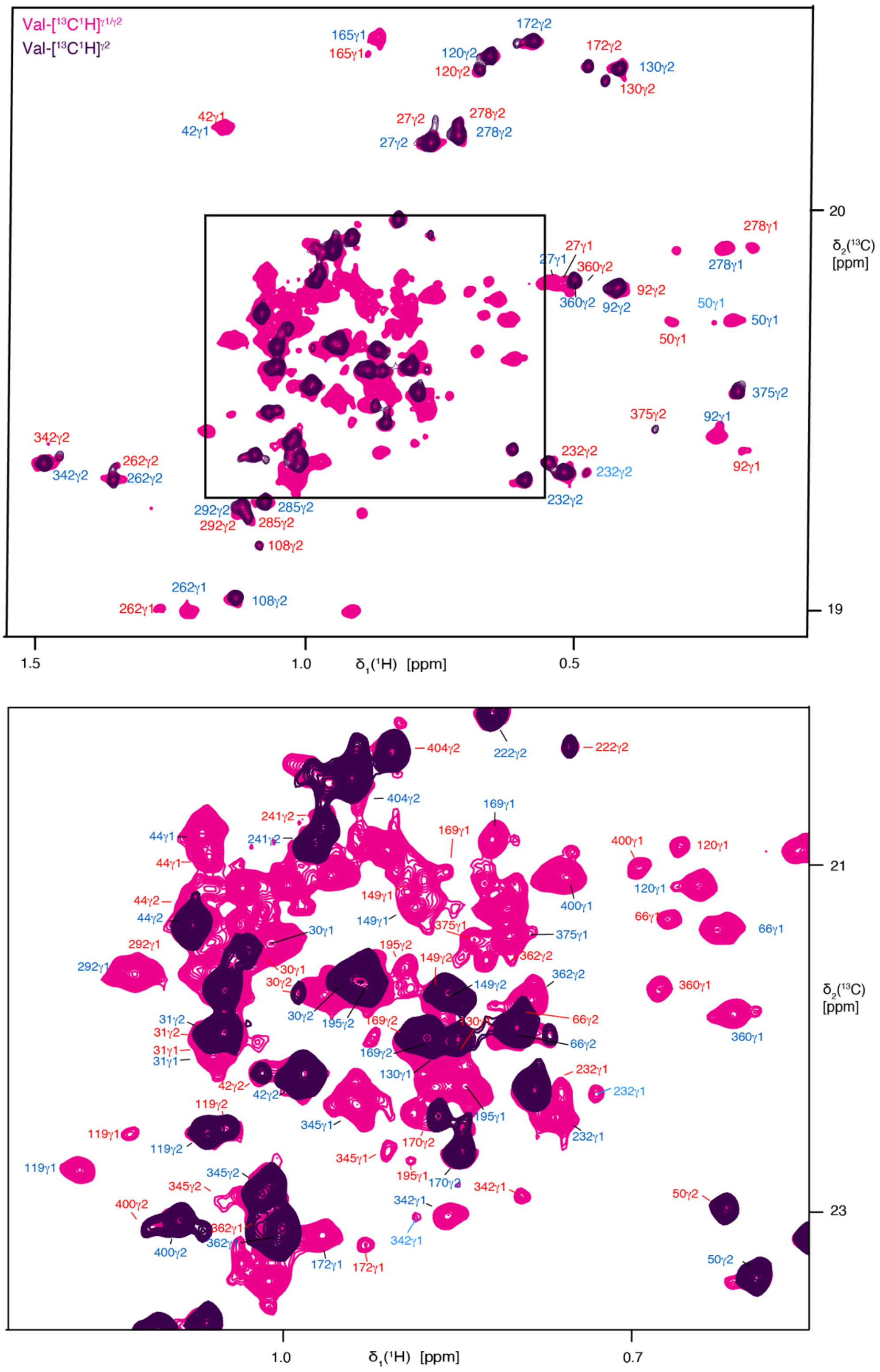

Supplementary Figure 6. Stereospecific assignment of BiP NBD Val-[^{13}C^{1}H]$^{\gamma1/\gamma2}$ in the ATP-bound state. 2D [^{13}C,^{1}H]-TROSY spectrum of methyl-labeled BiP NBD Val-[^{13}C^{1}H]$^{\gamma1/\gamma2}$ (pink) and Val-[^{13}C^{1}H]$^{\gamma2}$ (dark-pink) in presence of the ATP regeneration system (E1) but without E2. State- and sequence-specific assignments are indicated for the ADP-bound (light blue), ADP·Pi-bound (dark blue) and ATP-bound (red) states.

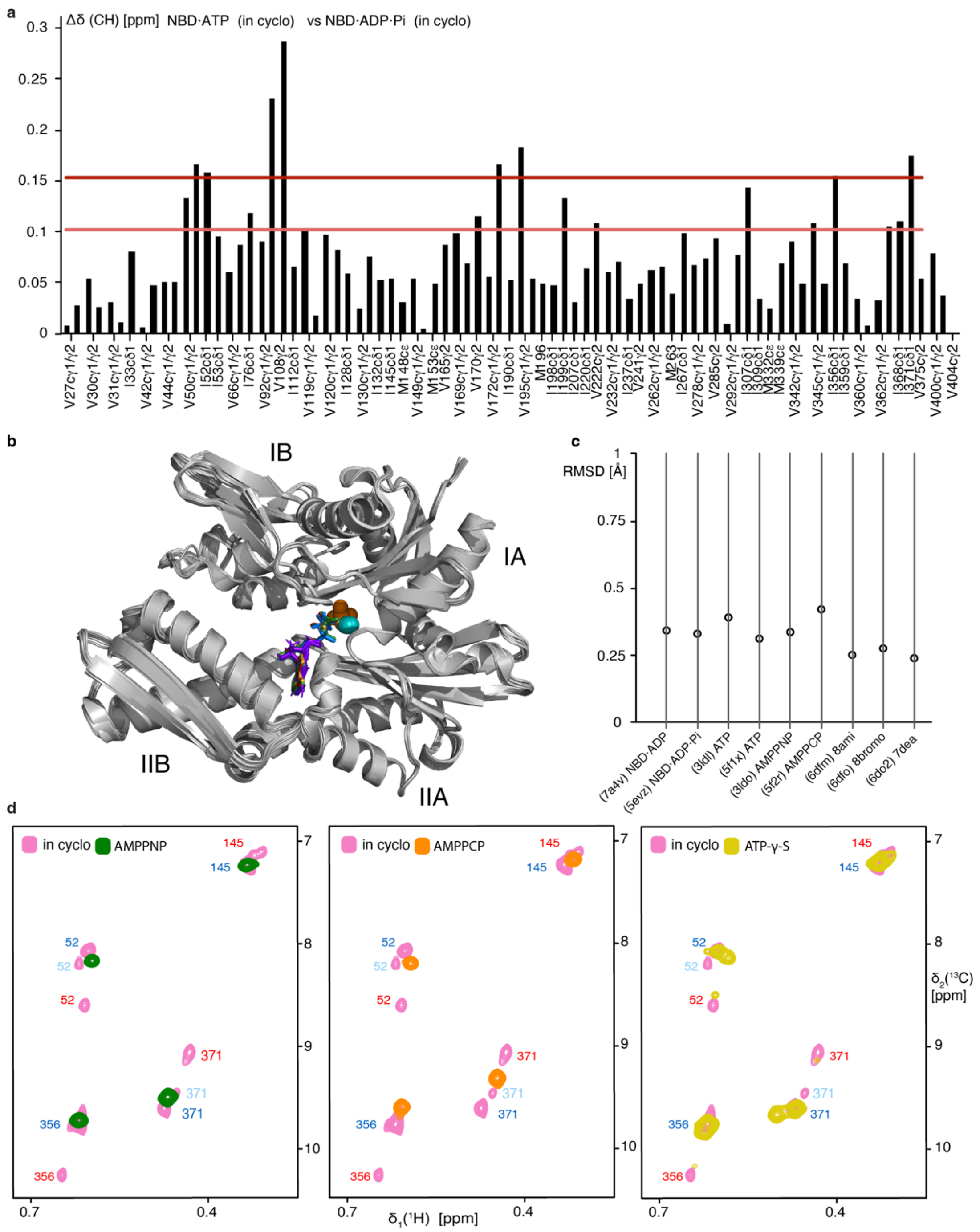

Supplementary Figure 7. Analysis of the BiP NBD ATP-bound state. a, Combined methyl chemical shift differences between the APD·Pi-bound and the ATP-bound state of BiP NBD in-cyclo. The magnitude of 1 and 1.5 SDs are indicated by an orange and a red line, respectively. **b**, Overlay of 9 crystal structures of BiP NBD in presence of different nucleotides (colored sticks). **c**, Backbone root means square deviation (RMSD - Å) between the structures shown in (b) and the NBD apo state (PDB 3LDN). **d,** 2D [^{13}C,^{1}H]-TROSY spectrum of methyl-labeled BiP NBD in equilibrium with the ATP analogs AMPPNP (green), AMPPCP (orange) or ATP-γ-S (yellow), overlayed with the spectrum in-cyclo (pink). State- and sequence-specific resonance assignments are indicated for the ADP-bound (light blue), ADP·Pi-bound (dark blue) and the ATP-bound (red) states in-cyclo.

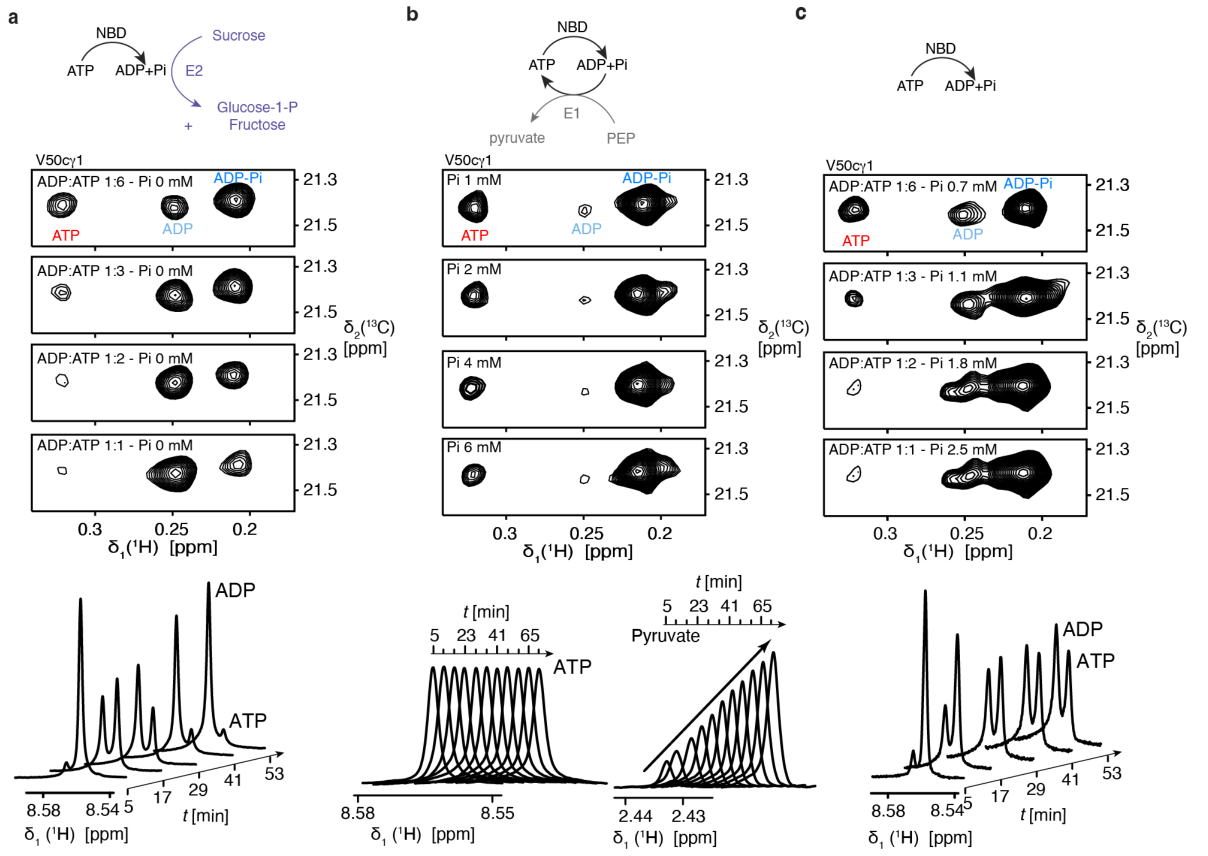

Supplementary Figure 8. The NBD functional cycle under different experimental conditions. a-c, Top panel: Scheme of the experimental setups. Middle panel: Sections of 2D [^{13}C,^{1}H]-methyl-TROSY spectrum of the methyl-labeled NBD in presence of the E2 (a), E1 (b) and no recycling system (c). The ADP:ATP ratio and phosphate concentration are indicated in the upper left corner of the spectra. Bottom panel: Series of 1D ^{1}H NMR spectra of the signals of ATP and pyruvate (b) and the signals of ADP/ATP (a,c). The time intervals are indicated next to the spectra, in (a,c) every other spectrum has been omitted to simplify the data visualization.

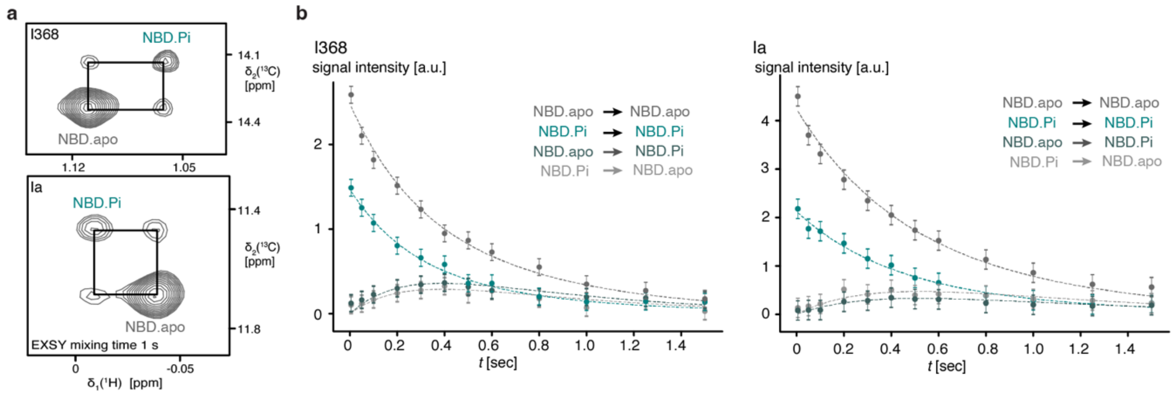

Supplementary Figure 9. NMR characterization of the NBD apo / NBD·Pi exchange. **a**, 2D [^{13}C,^{1}H]-TROSY-EXSY spectrum of methyl-labeled BiP NBD in equilibrium in presence of 200 µM phosphate at 25°C with an exchange delay of 1 s. Lines represent the connections between the NBD apo and NBD·Pi signals and signals corresponding cross-peak. **b**, Examples of fit of diagonal and transfer signals detected in 2D methyl-TROSY-EXSY experiments for the residues I368 and an unassigned isoleucine methyl group (Ia). The average fitted exchange constants, over three residues, between the NBD apo and NBD·Pi signals is k_{on}^{3} = 5 ± 2 mM^{-1} s^{-1} and k_{off}^{3} = 1.32 ± 0.31 s^{-1}.

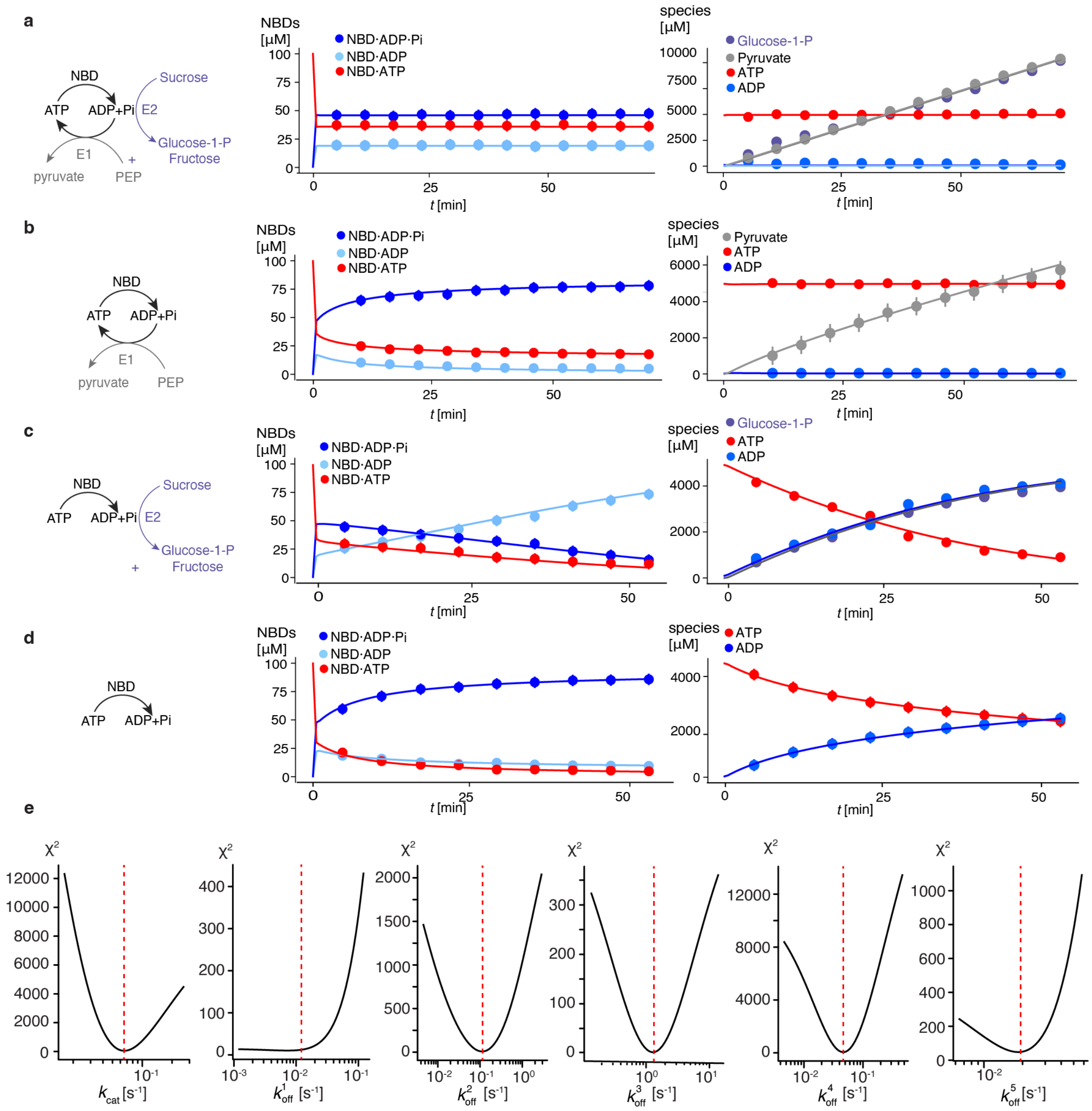

Supplementary Figure 10. Fitting of the functional cycle kinetic parameters. a-d, Global fit of the experimental data presented in the manuscript E1 +E2 (a), E1 (b), E2 (c), no recycling system (d). Data points are shown as circles NBD·ADP (blue), NBD·ADP·PI (dark blue) and NBD·ATP (red) (left) and pyruvate (grey), glucose-1-P (lavender), ATP (red) and ADP (blue) (right). The fit of the data is shown as lines in the same color as the data points. Data points and standard deviation for three independent experiments. **e**, Numerical stability of the global fitting of the experimental data.

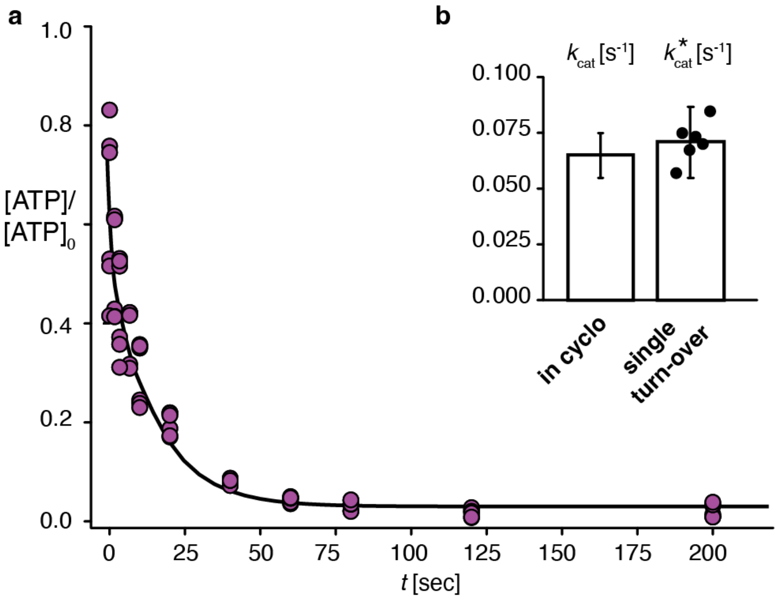

Supplementary Figure 11. Single turnover experiment matches the ATP lifetime in-cyclo. a, Single turn-over experiment[44] showing the decrease of ATP concentration over time for six independent experiments. The black line corresponds to the monoexponential fit of the data average. **b,** Comparison of the k_{cat} determined by in-cyclo NMR, and the k_{cat}^{*} determined in six independent single turn-over experiments. The bar represents mean and standard deviation.

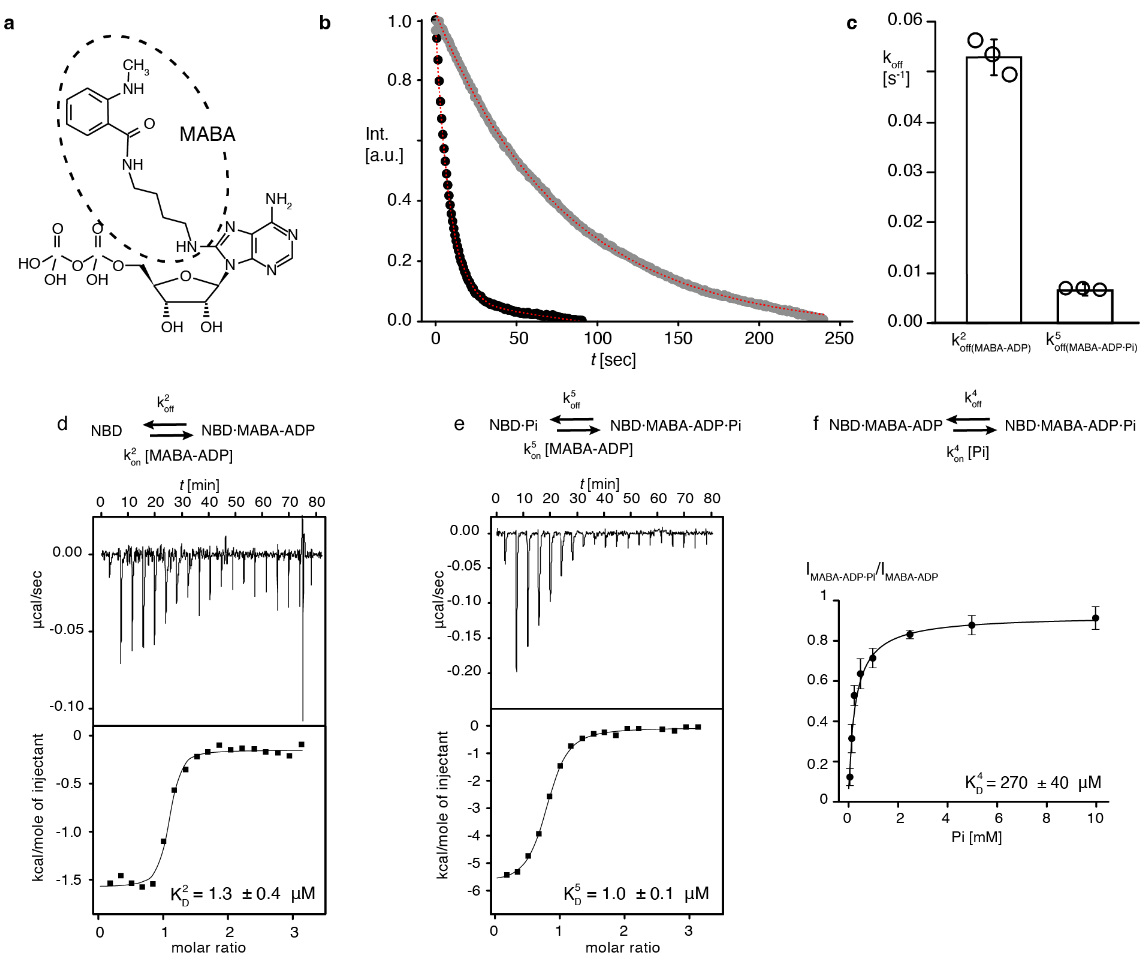

Supplementary Figure 12. MABA-ADP binding to the BiP NBD. a, Structure of the MABA-ADP molecule, the MABA fluorescent moiety is highlighted by a circle. **b**, Time courses of MABA-ADP dissociation from NBD without (black) or with 5 mM of Pi (grey): data points represent the mean for three independent experiments. The red lines correspond to the monoexponential fit of the data. **c**, $k^2_{off(\text{MABA-ADP})}$ and $k^5_{off(\text{MABA-ADP·Pi})}$ determined from the experiments presented in (b). Data points represent three independent experiments. The bar represents mean and standard deviation. **d,** ITC titration of 25 µM BiP NBD in the cell with 0.5 mM MABA-ADP in the syringe. **e**, ITC titration of 25 µM BiP NBD and 5 mM Pi in the cell with 0.5 mM MABA-ADP and 5 mM Pi in the syringe. **f**, NMR titration of 100 µM BiP NBD in the presence of 1 mM MABA-ADP with increasing concentration of Pi. The ratio of the population of the NBD·MABA-ADP·Pi and NBD·MABA-ADP bound state is presented. The data represents the average of eight independent NMR signals and the error bars the standard deviation. Data were fitted to extract the dissociation constant that are indicated next to the titration data for all the experiments.

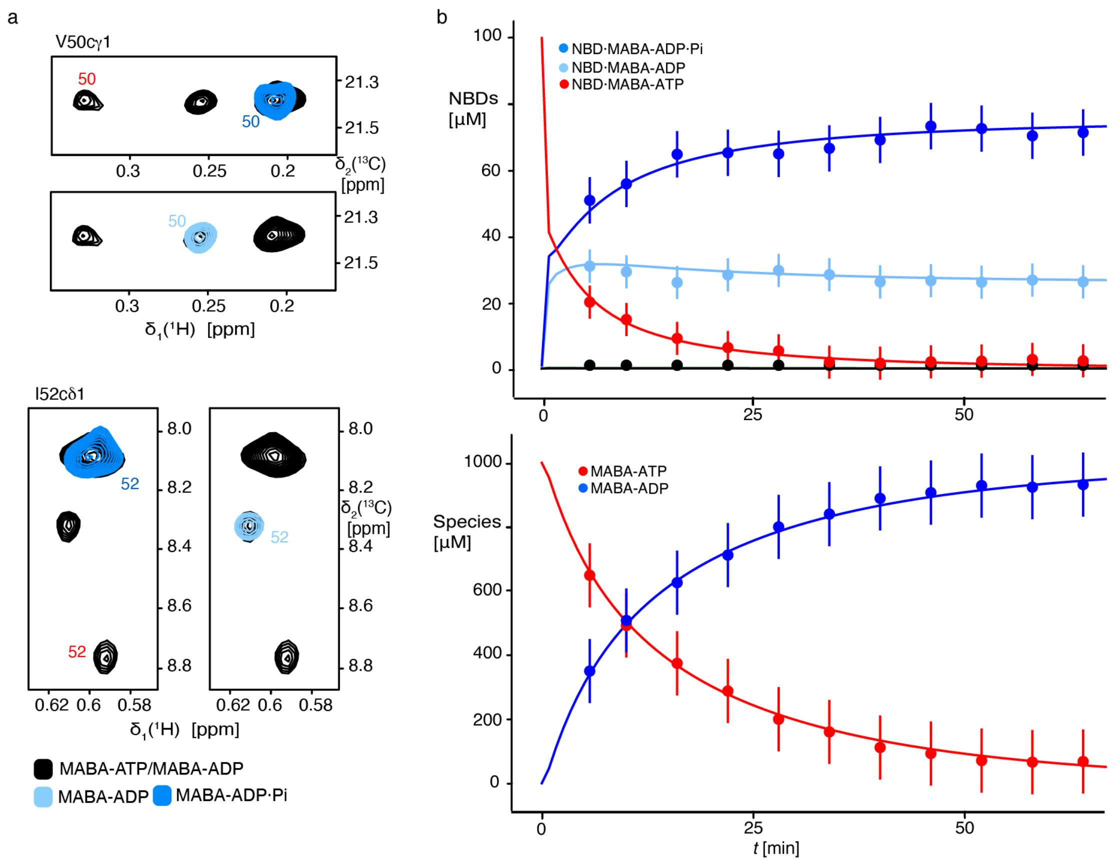

Supplementary Figure 13. Fitting of the MABA-ATP/MABA-ADP functional cycle kinetic parameters. **a,** Selected sections of 2D [^{13}C,^{1}H]-methyl-TROSY spectra of residue Val50γ1 and I52cδ1 at MABA-ADP:MABA-ATP ratio 1:3 overlapped with the equilibrium experiments of NBD with 1 mM MABA-ADP (light blue) and NBD with 1 mM MABA-ADP and 5 mM Pi (dark blue). **b**, Fitting of the MABA-ATP data in the absence of the ATP regeneration system. Data points are shown as circles for NBD·MABA-ADP (light blue), NBD·MABA-ADP·Pi (dark blue) and NBD·MABA-ATP (red) (top) and MABA-ATP (red) and MABA-ADP (blue) (bottom). The fit of the data is shown as lines in the same color as the data points. Data points and standard deviation for three independent experiments.

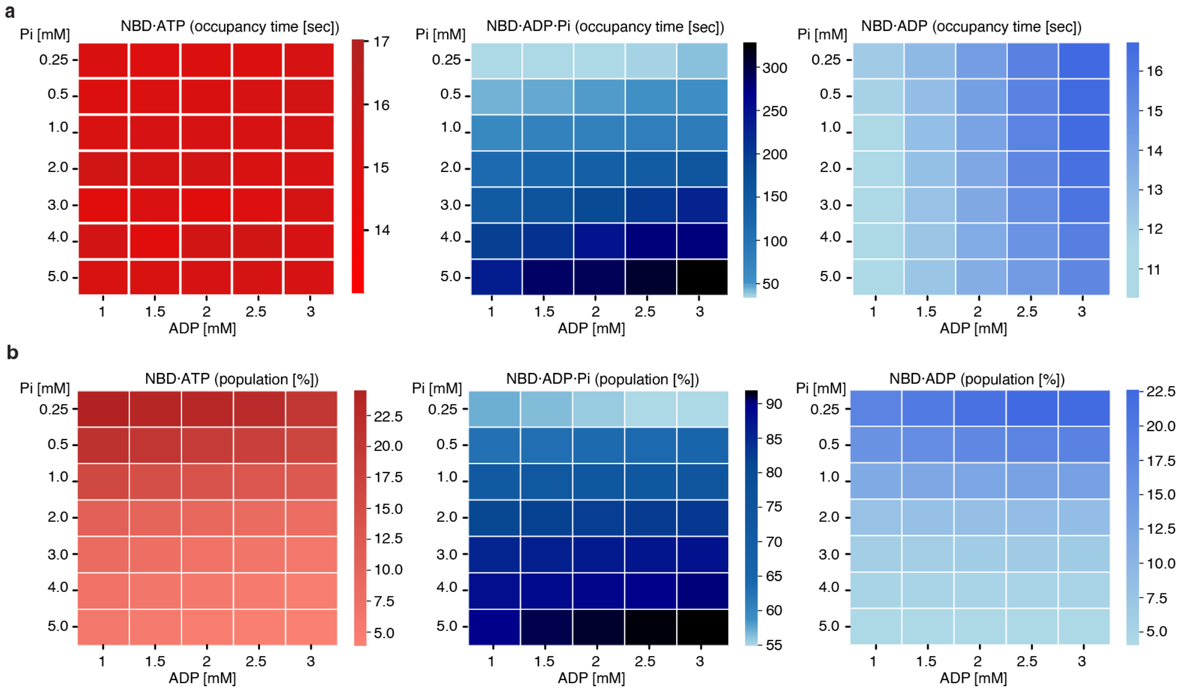

Supplementary Figure 14. The NBD functional cycle under physiological ADP and phosphate concentrations. a, Occupancy time of the three states of the NBD functional cycle NBD·ATP (red gradient), NBD·ADP·PI (dark blue gradient) and NBD·ADP (light blue gradient). **b**, Population of the three states of the NBD functional cycle NBD·ATP (red gradient), NBD·ADP·PI (dark blue gradient) and NBD·ADP (light blue gradient). The states NBD and NBD·Pi were never significantly present (< 1%) under any of the simulated conditions.

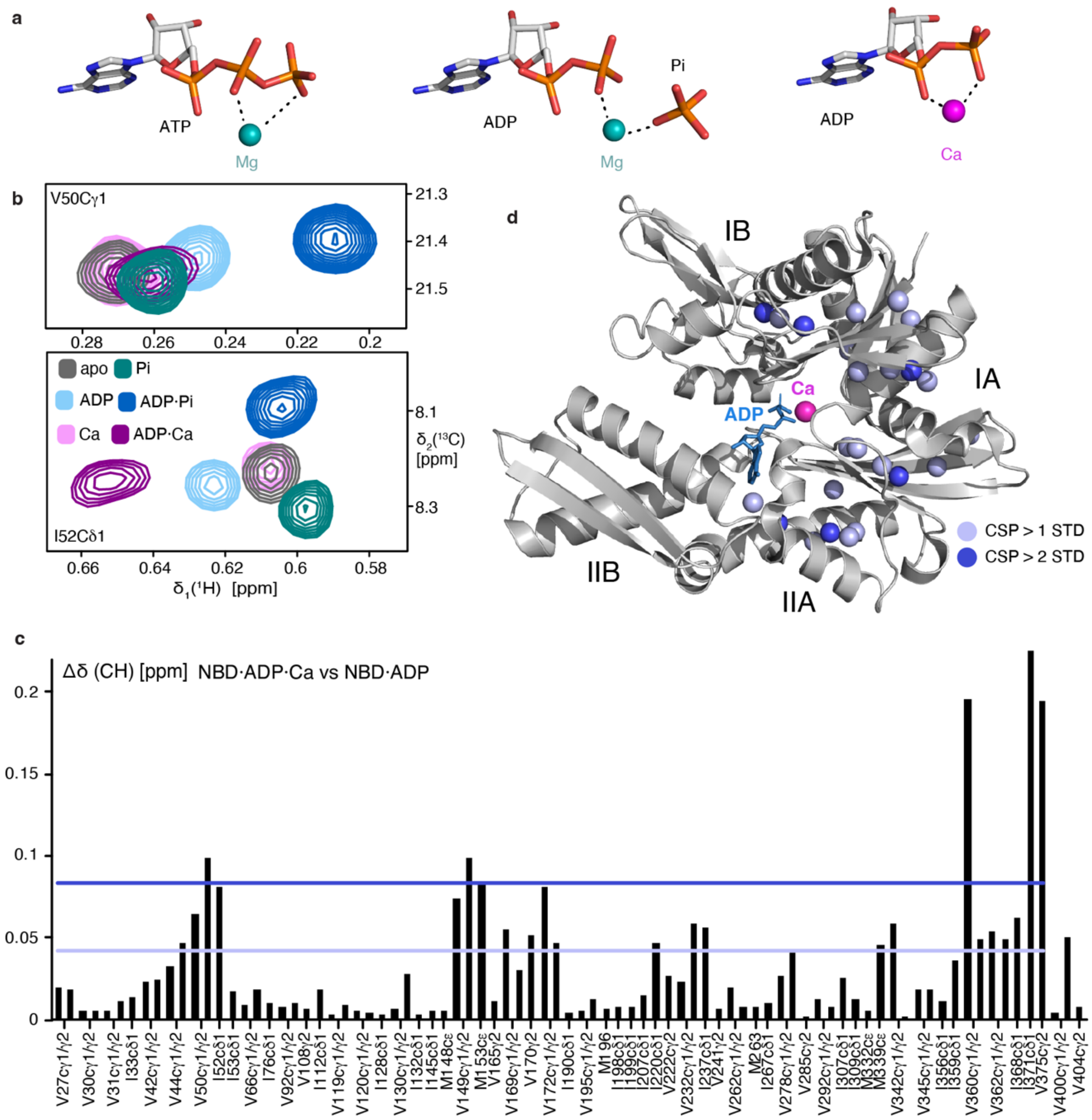

Supplementary Figure 15. Mapping of calcium binding to BiP NBD. a, Selected details from published structures of BiP. From left to right: yeast BiP(NBD)-Mg^{2+}-ADP (PDB 3QFU), human BiP(NBD)-Mg^{2+}-ADP·Pi (PDB 5EVZ), human BiP(NBD)-Ca^{2+}-ADP (PDB 6ZYH). **b,** Selected sections of 2D [^{13}C,^{1}H]-methyl-TROSY spectra of residue val50cγ1 or Ile52cδ1 for the equilibrium experiments of NBD apo (grey), NBD with 5 mM Pi (green), NBD with 1 mM ADP (light blue), NBD with 1 mM ADP·Pi (dark blue), NBD with 1 mM ADP-Ca^{2+} (dark-pink).and NBD with 3.3 mM calcium ($[Mg^{2+}]/[Ca^{2+}]$=3) (light-pink). **c,** Combined methyl chemical shift differences between the BiP NBD APD and the ADP-Ca^{2+}-bound state. The magnitude of 1 and 1.5 SDs are indicated by a light blue and a dark blue line. **d,** Structure of NBD in the ADP-Ca-bound state (PDB 6ZYH). Methyl groups with significant chemical shift upon calcium binding as identified in (c) are shown as spheres in light blue and dark blue, respectively. Color code as in (c).

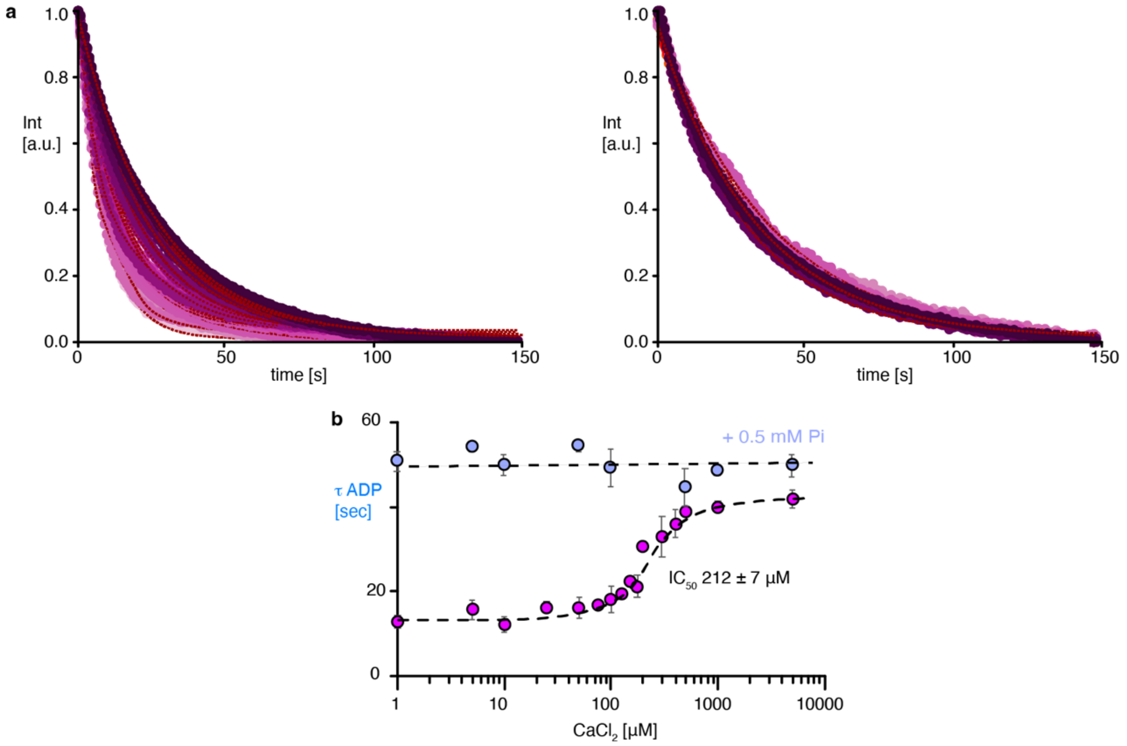

Supplementary Figure 16. Calcium binding to BiP NBD. a, Time courses of MABA-ADP dissociation from BiP NBD with increasing concentration of calcium 0.001 to 10 mM in the absence of Pi (left) and the presence of 0.5 mM Pi (right) (gradient light pink to dark pink): data points represent the mean for three independent experiments. The red line corresponds to the mono-exponential fit of the data. **b**, Mean-lifetime of the MABA-ADP-NBD complex at different calcium concentrations in the range from 0.001 to 10 mM, in the absence of Pi (pink circles) and with 0.5 mM Pi (blue circles). Data points represent the mean for three independent experiments and the error bar represents the standard deviation. The half maximal inhibitory concentration (IC_{50}) of Ca was calculated from three independent experiments

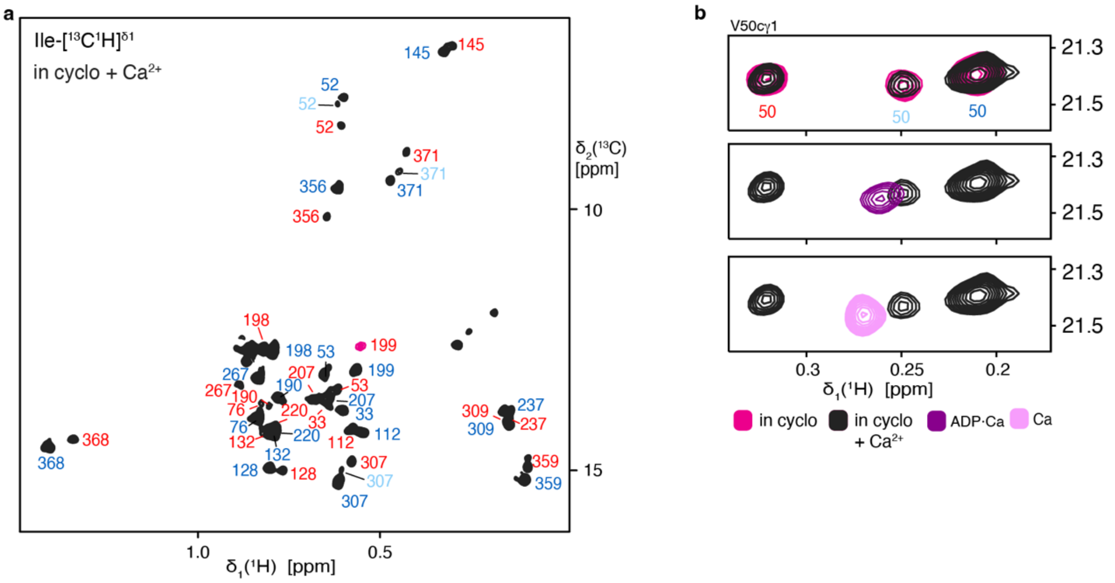

Supplementary Figure 17. Calcium does not affect the BiP NBD functional cycle. a, Section of 2D [^{13}C,^{1}H]-TROSY spectrum of methyl-labeled NBD in-cyclo and with 3.3 mM calcium ([Mg^{2+}]/[Ca^{2+}]=3). State- and sequence-specific resonance assignments are indicated for the ADP-bound (light blue), ADP·Pi-bound (dark blue) and ATP-bound (red) states. **b,** Selected sections of 2D [^{13}C,^{1}H]-methyl-TROSY spectra of residue Val50cγ1 in-cyclo with 3.3 mM calcium ([Mg^{2+}]/[Ca^{2+}]=3) (black) compared to in-cyclo condition without calcium (pink) and the equilibrium experiments of NBD with 3.3 mM calcium ([Mg^{2+}]/[Ca^{2+}]=3) (light pink), NBD with 1 mM ADP and 3.3 mM calcium ([Mg^{2+}]/[Ca^{2+}]=3) (dark pink).

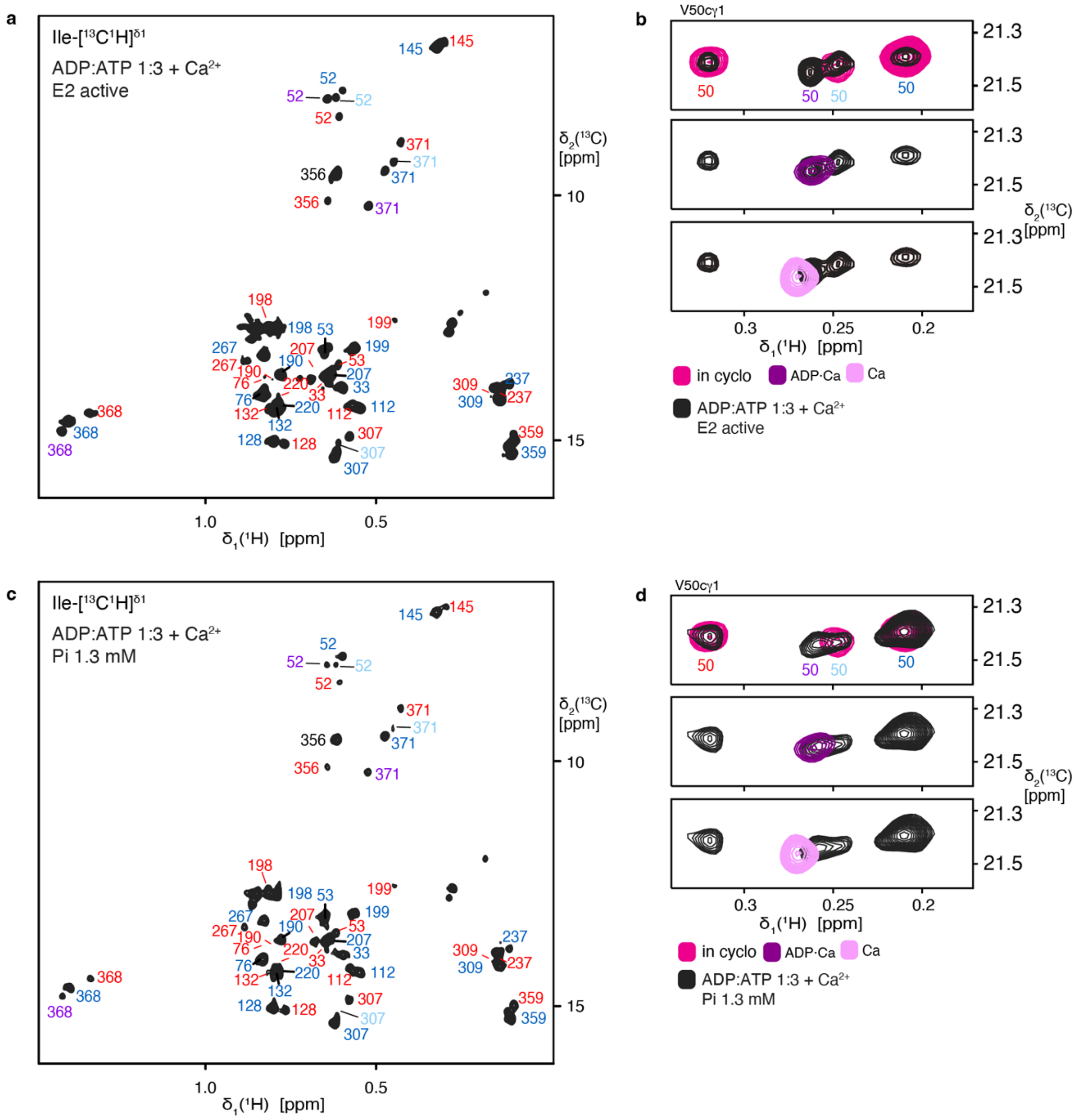

Supplementary Figure 18. NBD·ADP·Ca^{2+} forms as an off-cycle pathway state. a, 2D [^{13}C,^{1}H]-TROSY spectrum of methyl-labeled NBD in the presence of ADP:ATP 1:3, 3.3 mM calcium ([Mg^{2+}]/[Ca^{2+}]=3) and E2 active. State- and sequence-specific resonance assignments are indicated for the ADP·Ca^{2+}-bound state (dark-pink), ADP-bound (light blue), ADP·Pi-bound (dark blue) and ATP-bound (red) states. **b,** Selected sections of 2D [^{13}C,^{1}H]-methyl-TROSY spectra of residue Val50cγ1 in the presence of ADP:ATP 1:3 (pink) and with 3.3 mM calcium ([Mg^{2+}]/[Ca^{2+}]=3) (black) compared to in-cyclo no calcium (pink) and the equilibrium experiments of NBD with 3.3 mM calcium ([Mg^{2+}]/[Ca^{2+}]=3) (ligh-pink), NBD with 1 mM ADP and 3.3 mM calcium ([Mg^{2+}]/[Ca^{2+}]=3) (dark-pink). **c,d**, same as in (a,b) for the system in the absence of E2, *i.e* the phosphate concentration increases with ATP hydrolysis.
